# Prevalence of Hypertension and Its Associated Risk Factors in a Rural Black Population of Mthatha Town, South Africa

**DOI:** 10.3390/ijerph18031215

**Published:** 2021-01-29

**Authors:** Jyoti Rajan Sharma, Sihle E. Mabhida, Bronwyn Myers, Teke Apalata, Edward Nicol, Mongi Benjeddou, Christo Muller, Rabia Johnson

**Affiliations:** 1 Biomedical Research and Innovation Platform, South African Medical Research Council, Tygerberg, Cape Town 7505, South Africa; jyoti.sharma@mrc.ac.za (J.R.S.); sihle.mabhida@mrc.ac.za (S.E.M.); christo.muller@mrc.ac.za (C.M.); 2 Department of Biotechnology, Faculty of Natural Sciences, University of the Western Cape, Bellville, Cape Town 7535, South Africa; mbenjeddou@uwc.ac.za; 3 Division of Alcohol Tobacco and Other Drug Research Unit, South African Medical Research Council, Tygerberg, Cape Town 7505, South Africa; bronwyn.myers@mrc.ac.za; 4 Division of Addiction Psychiatry, Department of Psychiatry and Mental Health, Groote Schuur Hospital, University of Cape Town, Observatory, Cape Town 7925, South Africa; 5 Division of Medical Microbiology, Department of Pathology and Laboratory-Medicine, Faculty of Health Sciences, Walter Sisulu University, Mthatha 5117, South Africa; tapalata@wsu.ac.za; 6 Burden of Disease Research Unit, South African Medical Research Council, Cape Town 7505, South Africa; edward.nicol@mrc.ac.za; 7 Division of Health Systems and Public Health, Faculty of Medicine and Health Sciences, Stellenbosch University, Tygerberg, Cape Town 7505, South Africa; 8 Division of Medical Physiology, Faculty of Medicine and Health Sciences, Stellenbosch University, Tygerberg, Cape Town 7505, South Africa

**Keywords:** hypertension prevalence, body mass index, factors, hypertension treatment

## Abstract

*Background*: The occurrence of hypertension has been increasing alarmingly in both low and middle-income countries. Despite acknowledging hypertension as the most common life-threatening risk factor for cardiovascular disease (CVD), a dearth of data is available on the prevalence, awareness, and determinants of hypertension in rural parts of South Africa. The principal aim of the current study is to determine the prevalence and associated risk factors of hypertension among a black rural African population from the Mtatha town of Eastern Cape Province. *Methods*: This was a cross-sectional study, and individuals over 18 years of age were randomly screened using a World Health Organization stepwise questionnaire. Sociodemographic information, anthropometric measurements, fasting blood glucose levels, and three independent blood pressure (BP) readings were measured. Blood pressure measurements were classified according to the American Heart Association guidelines. Univariate and multivariate analyses were performed to determine the significant predictors of hypertension. *Results*: Of the total participants (n = 556), 71% of individuals had BP scores in the hypertensive range. In univariate analysis, age, westernized diet, education, income, and diabetic status, as well as overweight/obese status were positively associated with the prevalence of hypertension. However, in a multivariate logistic regression analysis only, age, body mass index (BMI), diabetic status, and westernized diet were significantly associated with a higher risk of developing hypertension. Gender, age, and BMI were potential factors having a significant association with the treatment of hypertension. Individuals who did not consider the importance of medicine had higher chances of having their hypertension being untreated. *Conclusions*: Prevalence of hypertension was high among the black rural African population of Mthatha town. Gender, age, westernized diet, education level, income status, diabetic as well as overweight/obese status were the most significant predictors of hypertension.

## 1. Introduction

Hypertension drives the global burden of cardiovascular disease and is a leading cause of cardiovascular-related mortality worldwide, with 1.39 billion affected adults and 10.4 million deaths globally [1,2,3]. The prevalence of hypertension has escalated globally with an estimated projection of a 30% increase in occurrence by the year 2025 [4]. Until recently, hypertension was acknowledged as a serious medical condition associated with mainly affluent regions of the world, however, the prevalence of this health condition has increased drastically with three out of four individuals residing in low and middle-income countries (LMICs) being hypertensive [5]. In Sub-Saharan Africa, hypertension has emerged as a major public health problem contributing to the rising number of premature deaths in this region. In particular, South Africa has been shown to have the heaviest burden of hypertension with an estimated prevalence between 27–58% [1,6]. Gutwatudde et al. [6] and later Gomez Olive et al. [7] reported that the prevalence of hypertension in South Africa will rise substantially if effective intervention strategies are not implemented timeously. These interventions include three key elements: identifying and addressing modifiable risk factors, diagnosis and screening of hypertension, and finally treatment with follow-up of diagnosed participants with hypertension. However, these interventions are suboptimally controlled in the rural parts of South Africa.

Indeed, if left untreated, hypertension can cause stroke, dementia, renal failure, blindness, myocardial infarction to name a few [3,8]. Despite being recognized as a major risk factor for cardiovascular disease, there is still a paucity of data available on the prevalence, awareness, risk factors, and control of hypertension among rural communities of South Africa. Information about trends and potential determinants of hypertension is essential for the improvement of community-based preventive strategies and management of hypertension.

Current evidence indicates that hypertension is a multifactorial condition influenced by many risk factors including genetic, sociodemographic, and behavioral factors [4,9,10,11]. Genetic and demographic factors including age, ethnicity, gender cannot be modified while behavioral factors such as physical inactivity, unhealthy dietary choices are often modifiable [12]. Socioeconomic status (SES), a sociological construct, refers to an individual’s relative position in the social hierarchy. Various indicators such as occupational group, educational attainment, level of income and wealth, and place of residence are utilized to measure socioeconomic status. Educational and socioeconomic status at the individual and parental level is reported to have an association with high blood pressure and awareness of hypertension [13]. Additionally, SES has been reported to predict health behavior and access to preventive health measures [14,15,16]. Various studies from African countries have shown an association between SES and hypertension but results are quite variable, with both positive and negative associations being reported [8,17,18]. Some studies have also investigated the influence of body mass index (BMI), physical activity, and diet on blood pressure patterns. Body mass index has been directly associated with a higher risk of developing hypertension while physical activity is inversely associated with hypertension in developed economies, however, findings were not consistent in low to middle-income countries [19,20,21,22]. Furthermore, high BMI (overweight/obesity) is often associated with insulin resistance and has important implications for the development of type 2 diabetes mellitus (T2DM) [23,24] that is a growing problem across Africa affecting 19 million adults including 14.5 million in Sub-Saharan Africa [25]. Both obesity and T2DM are also linked to higher mortality risks due to cardiovascular diseases [26,27].

These biological and behavioral factors are demonstrated to have an uneven distribution across socioeconomic strata which makes them possible mediators of the observed association between sociodemographic variables and blood pressure [28]. These sociodemographic and clinical factors not only determine the prevalence of hypertension but also have a drastic impact on the control of hypertension. This was first observed by Adeniyi et al. [9] who reported poor control of hypertension in individuals living with T2DM in the town of Mthata in South Africa. Despite the availability of advanced diagnostic options and several therapeutic drugs, various studies have reported suboptimal BP control in rural and urban settings of SA [3,29,30,31,32]. A large proportion of hypertensive patients remain unaware of their condition and do not receive any treatment. The primary health care system of South Africa is overburdened with ongoing infectious disease challenges along with the rising demands from increasing hypertension and related complications with limited resources available [33]. As such management and control of hypertension are critical in countries like South Africa, a country with extreme levels of poverty and socioeconomic inequality because of its apartheid history [34]. Information on the prevalence and factors associated with hypertension among various communities is urgently needed for the development of community-specific hypertension preventative strategies.

The scarcity of data on sociodemographic, bio-behavioral, and comorbidities associated with hypertension in rural areas of South Africa raises the need for research among rural populations for developing effective and population-based interventions for the prevention of hypertension. The objective of this study, was, therefore, to determine the prevalence, treatment, determinants, and associated comorbidities of hypertension among the rural population of the Eastern Cape, South Africa. Such epidemiology data is urgently required by health policymakers to tailor specific strategies for the control of hypertension.

## 2. Material and Methods

### 2.1. Study Design

The present cross-sectional study included the black African population from four districts (OR Tambo, Alfred Nzo, Chris Hani, Joe Gqabi) with five sub-districts and fourteen community health care centers in the Eastern Cape province, South Africa. This province is the second largest province in the country and serves a population of 7,130,480.

### 2.2. Eligibility Criteria

To be eligible for this study, patients needed to be over 18 years of age and belong to African ethnicity. Exclusion criteria were pregnant women and reluctance to participate in the study.

### 2.3. Ethical Approval

The study conforms to the ethical guidelines of the Declaration of Helsinki [35] and obtained approval from ethics committees of Water Sisulu University (073/15) and South African Medical Research Council (EC028-8/2020). The participants were provided an information sheet written in both English and IsiXhosa languages with details of the purpose, the process of research, rights of the participants, and details of the contact person for any inquiry before granting a written informed consent.

### 2.4. Sample Size and Sampling

The appropriate sample size was estimated using the following formula:

$$n=\frac{p\left(1-p\right)z^{2}}{d^{2}}=\frac{0.30\left(1-0.30\right)1.96^{2}}{0.05^{2}}=322$$

where *z* is the confidence level, p is the expected proportion of patients with hypertension and d is the margin of error. *p* was set at 0.30 and the desired precision is 5%. The calculation was done at a 95% confidence level. A total of 556 participants were included just to compensate for incomplete records.

### 2.5. Sampling Procedure

A total of 556 participants were randomly selected in series at the hospital outpatient department (without having prior knowledge of their hypertension status). However, 19 participants were not included in the further analysis due to incomplete data.

### 2.6. Data Collection

A face-to-face interview was conducted, and informed consent was obtained from all the participants and after consenting, all respondents were medically examined by trained survey staff. Consent forms and the World Health Organization (WHO) STEPwise questionnaire were uploaded onto the Research Electronic Data Capture (REDCap) a web-based application for building and managing online surveys and databases [36]. The questionnaire included information on gender, age, race, marital status, level of education, monthly income, the status of employment, and behavioral characteristics (physical activity, dietary intake, knowledge, and beliefs of hypertension, and its treatment). Additionally, information about anthropometric measurements (weight and height), was also included. A pilot study was performed on 180 participants (not included in the study) to ascertain the validity of the instrument.

### 2.7. Assessment of Overweight/Obesity

Participants were weighed bare feet with light clothing to the nearest 0.1 kg using a standard beam balance. Height was measured to the nearest 0.1 cm on the mounted Stadiometer, and then BMI was calculated. Participants were categorized into normal, overweight, and obese according to the WHO standards [37]. Participants were considered as overweight if BMI was 25–29.9 kg/m^2^ and were categorized as obese if their BMI was ≥ 30.0 kg/m^2^. Participants with BMI ≤ 25 were either classified as normal or underweight.

### 2.8. Diagnosis of Type 2 Diabetes Mellitus (T2D)

A blood sample was taken after an overnight fast. The diagnosis of T2D was confirmed according to the 2011 American Diabetic Association criteria [38]. Participants were categorized as normal (fasting blood sugar level < 5.6 mmol/L), prediabetic (fasting blood sugar level between 5.6–6.9 mmol/L), and diabetic (fasting blood sugar level >7 mmol/L).

### 2.9. Measurement and Definition of Blood Pressure

Blood pressure was measured using a slight touch ST-401 blood pressure monitor (Nobel Supplies Group, NYC, USA) according to the standard operating procedures. Before the measurement, patients were asked to rest for five minutes with the arm at the level of the heart and the feet together. Screening and diagnosis of hypertension (HTN) were defined as per guidelines provided by the American Heart Association (AHA), 2018 [39]. A mean of three repeated BP measurements was used in all calculations and analyses. Thereafter, participants were categorized into three categories based on their BP: (1) elevated blood pressure (systolic blood pressure (SBP):120–129 mm Hg and diastolic blood pressure (DBP) < 80 mm Hg), (2) HTN grade 1 (SBP:130–139 mm Hg and DBP:80–89 mm Hg), and (3) HTN grade 2 ≥ 140 mm Hg and ≥ 90 mm Hg. Each subject was asked questions on the awareness and treatment of hypertension. Clinical records of participants identified as hypertensive were assessed for three months post initial diagnosis to confirm the disease state.

### 2.10. Socioeconomic and Environmental Variables

Information about sociodemographic and environmental variables was collected during personal face to face interviews using a WHO stepwise questionnaire uploaded on REDCap. Age (years) was considered as a continuous variable, race was self-reported by participants as per historical group categorization in South Africa.

Four self-reported sociodemographic factors were assessed: level of education, employment, income, and marital status. Education variables were recorded as one of four categories from no schooling to primary school (category 1), high school (category 2), and tertiary (category 3). Individuals were categorized as employed or unemployed based on their employment status. Participants were defined as unemployed if they did not have any occupation in either the formal or informal sectors. The income group was categorized into <R1000 ($65) and ≥R1000 ($65) according to the total monthly amount earned/accruing to an individual’s household. Marital status was recorded as single or in a relationship.

Self-reported levels of physical activity were measured and categorized into ≥150 min per week or <150 min based on the WHO recommendations [40]. The dietary intake of participants was obtained through the 24-h recall method and the information was used to describe adherence to the westernized diet. Westernized diet is defined as a diet rich in saturated fats, refined grains, sugar, and salt with reduced consumption of fruits and vegetables [41]. Diet was further categorized into three categories: (1) low intake of westernized diet referred as a diet low in salt, refined grains and high in veg/fruit or with only one of the risk factors; (2) moderate intake of a westernized diet with two of the risk factors; and (3) high intake of westernized diet, with all three of dietary risks present.

### 2.11. Data Analysis

Statistical package for social sciences (SPSS) windows version 16.0 (SPSS, Inc., Chicago, IL, USA) was used to analyze the data. The unadjusted odds ratio was utilized to calculate the association between hypertension and socio-economic demographic factors. Multivariate regression analysis was carried out between dependent (hypertension) and independent variables (socio-economic and demographic). Significance was tested at 95%, *p* < 0.05 was taken as significant.

## 3. Results

### 3.1. Participant’s Sociodemographic and Clinical Characteristics and Levels of Blood Pres

**Figure 1:**
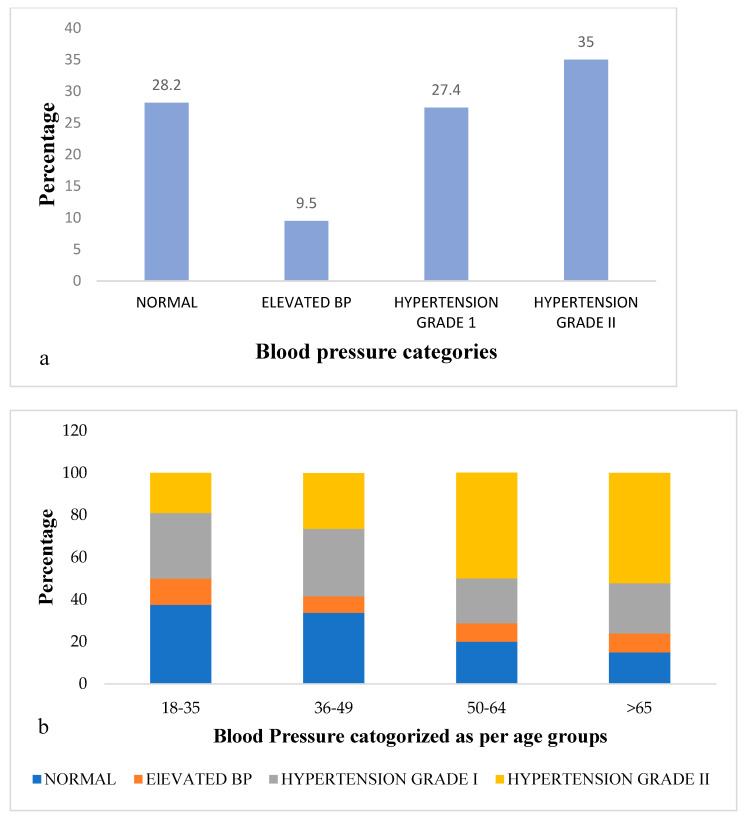
Distribution of participants according to their Blood pressure.

The study sample included 556 black African individuals over 18 years of age, and women comprised the bulk of the sample (85.1%). Table 1 provides information about the sociodemographic and clinical characters of the participants along with the status of their blood pressure. Three-quarters of the participants (71.0%) had blood pressure readings in the hypertensive range (Table 1). More than one-third of participants had BP readings that met the criteria for hypertension grade II and almost half of them belong to the elderly age group (>50 years of age; Figure 1a,b). However, only 44.3% of the total participants with BP in the hypertensive range were aware of their hypertensive status.

Overall, participants were predominantly single (61.1%), unemployed (56.1%), and with limited monthly income (58.9% earned < R1000). In terms of education, 52.9% of participants had completed high school, with only 17.7% progressing to post-school education. In terms of clinical characteristics, almost three-quarters of participants were either overweight or obese and about 40% were categorized as prediabetic or diabetic. More than half of the participants were physically inactive and followed a westernized diet with a high intake of salt and refined carbohydrates and a low intake of fruit and vegetables. Furthermore, almost 82% of individuals with increased consumption of a westernized diet were hypertensive. Around 84.5% of the hypertensive participants considered that hypertension cannot be controlled with medication, though most of them (70.6%) were hypertensive. Only 22.3% of the participants considered that hypertension can be controlled with diet.

Table 1 shows significant associations of age (*p* < 0.001) westernized diet, as well as income (0.039), diabetic and overweight/obese status with the prevalence of BP scores in the hypertensive range. However, other variables such as education (*p* = 0.215), employment status (*p* = 0.948), physical activity (*p* = 0.361), hypertension beliefs among participants such as the importance of medicine (*p* = 0.342), and dietary control (*p* = 0.969) did not show any significant association with the BP scores in the hypertensive range. However, gender demonstrated a marginally significant association (*p* = 0.061) with BP scores.

Variables associated with hypertensive status at *p* < 0.1 were entered into a multivariate logistic regression model (Table 2). In this model, the only variables that remained significantly associated with hypertensive status were age, BMI, diabetic status, and the westernized diet. Specifically, participants between 50–64 years of age [*p* = 0.039, aOR 1.87 (1.03–3.39)], and ≥65 years of age [(*p* = 0.009, aOR 3.20 (1.34–7.63)] had almost doubled and more than tripled the odds of being hypertensive, respectively relative to the participants of 35 years of age or younger. Individuals who were obese also had more than tripled the odds of being hypertensive relative to those with a normal BMI (*p* ≤ 0.001, aOR = 3.52; (2.01–6.18)]. Subjects with a blood glucose reading indicative of diabetes had more than doubled [(*p* = 0.034, aOR = 2.24; 1.06–4.72)] the odds of being hypertensive as compared to those with normal blood glucose readings. Finally, participants who were moderately or highly adherent to a westernized diet with a high intake of salt, refined carbohydrates, and low intake of fruit and vegetables also had significantly greater odds [(*p* ≤ 0.001, OR = 5.35, 2.85–10.05)] of being hypertensive. Additionally, high income (≥R1000) also indicated to increase the likelihood of hypertension though with the borderline significance [(*p* = 0.087, OR = 1.47; 0.95–4.29).

### 3.2. Potential Variables Affecting Treatment Status of Hypertension

Among hypertensive participants, we then explored potential factors that were associated with untreated hypertension (Table 3).

The given variables such as gender (*p* = 0.002), age (<0.001), and education (*p* = 0.052) (Table 3) illustrated a significant association with treatment status among hypertensive participants. However, relationship status, unemployment, income level, diabetic status, physical activity, and westernized diet pattern of the participants did not have any significant association with the treatment status. Interestingly, although knowing the importance of medication did not have any significant association with BP scores in the hypertension range, however, this variable certainly had been observed to have a significant association with the treatment of hypertension. In Table 3 multiple logistic regression analysis has also been utilized to investigate the significant predictors of hypertension being untreated.

It is indicated that being male increases the risk of being untreated [(*p* = 0.034, OR 2.93 (1.90–7.95)]. However, participants who belong to the age group 50-64 [(*p* = 0.035, OR 0.45 (0.22–0.95)] and the age group ≥ 65 [(*p* = 0.000, OR 0.190 (0.07–0.48)] had lower risks of being untreated. It has been observed from the odds ratio that being overweight [*p* = 0.109, OR 0.51 (0.23–1.16)] and obese [(*p* = 0.002, OR 0.30 (0.15–0.64)] lowers the odds of being untreated. Similarly, those who did not understand the value of medication and its role in hypertension management had higher odds of being untreated [(*p* = 0.026, OR 3.02 (1.5–6.1)].

## 4. Discussion

Hypertension is a predominant marker of complex vascular diseases and is a serious public health threat in South Africa [7,42]. Additionally, it is an independent and preventable risk factor for all causes of premature deaths [43]. The burden of hypertension is further exacerbated by the limited information available on the prevalence, treatment, and potential determinants of hypertension in rural communities. Such epidemiological data on the prevalence and factors associated with hypertension are urgently required for the development of community-based interventions. This study, therefore, aimed to evaluate the prevalence and the biological and sociodemographic variables of hypertension among the residents of Mthatha, South Africa.

The current study not only reports on the associated factors of prevalence but also assesses the potential predictors determining the treatment status of hypertension. The high prevalence of hypertension (71%) among residents of rural communities is consistent with the previously reported prevalence in the South African Demographic and Health Survey for non-urban black South Africans [34,44]. However, previous studies from rural areas of Sub-Saharan Africa have reported lower rates of hypertension prevalence that ranges from 5%–52% [45,46,47]. Nonetheless, the prevalence of hypertension in South Africa is reported to be 30.4% as per the recent South African National Health and Nutrition Examination Survey (SANHANES) [48,49,50]. As such, results presented in the current study confirmed previous findings and also highlight a serious concern of the rising prevalence of hypertension in rural South Africa [48,51,52,53].

Various studies have indicated that in comparison to the developed countries, developing countries are experiencing a higher rise in prevalence rates [54,55,56] without any further improvement in awareness and control rates. This trend is not surprising considering urbanization, unhealthy lifestyle and dietary habits, and their consequent adverse health effects on the health of the population. Furthermore, limited health services due to inadequate funds, poor infrastructure, lack of equipment compounded with medical illiteracy are major obstacles for preventing and controlling hypertension. Likewise, with South Africa being a middle-income country, results from this study supports the view that there is an epidemiological transition of non-communicable diseases to low- and middle-income countries including their rural population.

Moreover, the influence of gender on the risk of developing hypertension has not been well documented with contradictory results being reported regarding the association between gender and prevalence of hypertension. Most findings conducted in different districts of South Africa reported females having a lower prevalence of hypertension than males [57,58,59,60,61,62]. In contrast, Alberts et al. [63] and later Mkhonto et al. [64] showed that the prevalence was low among males. The current study is consistent with these studies, reporting a higher prevalence of hypertension among females than males.

Additionally, many studies have demonstrated an association between age and risk of developing hypertension with hypertension being more prevalent among older people [52,57,65]. These findings were also supported by the World Health Organization (WHO, 2012) reporting that 75% of adults aged > 50 years of age are hypertensives in South Africa [15]. The results of this study also confirmed that age is a significant predictor of higher HTN prevalence. Participants belonging to 50–64 years and of >65 years of age had a higher risk of having hypertension in comparison to the young participants. It has been suggested that an increase in vascular resistance contributes to an increase in hypertension in elderly people [66,67]. Most chronic diseases occur during this stage of life because of the interactions between multiple disease processes and loss of physiological functions [68].

Zhou et al. [69] and Mashiane et al. [70] reported a high prevalence of overweight and obesity in South African rural populations in all age groups. Similarly, our study also revealed a high prevalence of obesity in the studied rural population. Previous studies suggested that higher BMI is a major risk factor for developing hypertension [43,57,71] and there is a linear relationship between an increase in BMI and hypertension [39]. Additionally, it has also been demonstrated that obesity and visceral adiposity has been positively correlated with the renin-angiotensin system that controls blood pressure [72,73,74].

Furthermore, It has also been reported that there is a substantial overlap between etiology of hypertension and diabetes because of their common metabolic pathways and shared risk factors [75]. In a previous study [9] from the same area, a high prevalence of hypertension (81%) among individuals with type 2 diabetes has been reported. Another study reported that individuals with diabetes have almost double the chances of developing hypertension than those without diabetes [76]. In addition to this, a positive association has been identified between obesity, diabetes, hypertension, and cardiovascular disease [77]. Concerning the biological determinants of hypertension, the current study agrees with the previous findings of associating obesity and diabetes with an increased risk of developing hypertension. A high prevalence of non-communicable diseases (NCDs) such as hypertension, obesity, diabetes, and other cardiovascular diseases in urban and rural parts of South Africa is a major public health concern because of the serious added economic burden with these diseases [77,78]. Considering epidemiological and nutritional transition in Sub-Saharan Africa, the World Health Organization has also projected that Sub-Saharan African countries will experience a high prevalence of cardiovascular diseases in the next decade [79,80].

Sociodemographic variables such as education, employment, household income, and household assets have also been reported to influence blood pressure levels [28,81,82]. Some studies have revealed an inverse relationship between socioeconomic status and prevalence of hypertension [83] and have indicated that the risk of cardiovascular disease tends to be higher among individuals of low socioeconomic status than those belonging to a higher socioeconomic status [84,85]. Regarding sociodemographic variables, the current study reports that relationship status, education, and employment of participants are not significant predictors of hypertension. However, the income level is demonstrated to have a positive association with the prevalence of hypertension. In a multivariate logistic regression analysis, participants with a higher income level (≥R1000) are more likely to be at increased risk of hypertension with borderline significance reported. It could be due to the different dietary habits of the two groups. These findings could also be explained by the fact that higher socioeconomic status does not always relate to a better nutritional status of a population but could lead to inappropriate nutritional patterns which may predispose them to the development of cardiovascular disease [86]. Furthermore, it has also been shown that higher income groups may be at higher risk of developing hypertension due to the high consumption of processed foods along with their sedentary lifestyle [87]. Contradictory findings between the current study and previously reported studies could be due to the different measures of socioeconomic status (SES) in different settings. Individuals with low SES may not be able to buy expensive healthy foods or limited access to health resources [88].

The high consumption of the westernized diet is likely to have an adverse impact on the health of the South African population. These findings have also been reported in other African populations [3,89,90,91,92]. This trend is mainly because of globalization and increasing adherence to the westernized diet that has been reflected in the rapid expansion of fast-food restaurant chains in semi-urban and rural settings of South Africa and Sub-Saharan Africa [93]. Our study also reports that a high proportion (about 88%) of the studied population consumed moderate to high amounts of westernized diet. Further, multivariate regression analysis showed that moderate to high consumption of westernized diet was associated with an increased risk of developing hypertension.

There is very limited information available about the awareness, treatment, and several other predictors of hypertension treatment in South Africa. Various studies [94,95] have emphasized the importance of a better understanding of awareness, treatment, and control for improving the management of hypertension. This is critical to understand and address barriers in the treatment of hypertension, therefore, in this study potential determinants of hypertension treatment were also investigated. Being male was associated with higher odds of untreated hypertension. This could be due to better utilization of health facilities by women as suggested by previous studies [93,96]. It was assessed that being male tripled the chances of being untreated than females. These findings agree with another study that demonstrates that women may be better receptive to the treatment and have regular visits to health care facilities [97]. Likewise, another study by Adeniyi et al. (2015) also agrees that females are more likely to visit health care facilities [93]. Traditional cultural factors in Africa could also be responsible for the underutilization of health facilities among males; being the breadwinners, males hardly go to the health care facilities unless they are sick. [98,99].

It is evident from various studies that education is a vital component of health and education elements should always be included in public health promotion and for the reduction of health disparities [100,101]. Various studies have suggested that lower education level influences the treatment and control of hypertension through limited knowledge of disease preventive measures, unhealthy diet, psychosocial stress due to hazardous occupations [102,103].

In contrast, this study showed that higher education level seems to increase the odds of being untreated however, only high school education was a significant predictor when compared to primary level education. The relationship between education and health is quite complex, it is generally assumed that an educated person will be more knowledgeable about health aspects and receptive to new drugs and treatment [104,105]. However, these observations could vary in different races and environments; the population in the current study is poor, might not have quality education and better access to health facilities. Moreover, the association was non-significant in multivariate analysis, therefore a greater sample size is required to make any conclusion.

Additionally, it has been suggested that overweight/obese individuals are more likely to have their blood pressure documented as compared to normal-weight individuals and recoding of blood pressure measurements could be associated with the treatment. Moelnaar et al. [106] and Rose et al. [107] reported that obese individuals are more likely to receive hypertension treatment. Likewise, the current study also reveals that being overweight/ obese reduces the odds of being untreated than healthy individuals.

Successful implementation of health programs relies on the knowledge, perceptions, and beliefs of any community; only a very few studies in South Africa have focused on the existing information that the community has on hypertension [108,109,110]. Therefore, in the current study information was also taken about patients’ beliefs in controlling hypertension with medicine and the importance of dietary control. A small proportion of participants valued medicine for the treatment of hypertension and this belief did not have any significant association with having hypertension however, participants who do not give value to the use of medication for the treatment of hypertension, had higher odds of having untreated hypertension. Beliefs about illness and medicines are interrelated and may influence compliance. Beliefs of medicine and hypertension are not only predictive of compliance but also important in achieving concordance and could be a target for achieving interventions to improve compliance [111].

Current findings in the poorly resourced area of the country reflect the poor health status of individuals living with NCDs and highlight the threat of existing vascular disease. Therefore, demand urgent action in terms of prioritizing quality health services to rural communities.

## 5. Conclusions

Our results suggest that the prevalence of hypertension was high among the black rural African population of Mthatha Town. It is revealed that age, income, westernized, higher levels of blood glucose and BMI are positively associated with hypertension. These findings also highlight the changing patterns of dietary habits among the rural communities of South Africa. Additionally, it has also been demonstrated that gender, age, education, BMI, and belief in controlling hypertension with medication are key predictors of determining the treatment status of hypertension. This study showed that about half of the participants were unaware of their hypertension status and as such highlights the urgent need for hypertension education, screening, and control within the Mthatha area.

### Strengths and Limitations of the Study

The current study addresses the problem of hypertension in the rural community of Mthatha town in Eastern Cape Province, South Africa. It highlights the critical requirement for policymakers to recognize the potential determinants of hypertension and its treatment status for the management of hypertension in various communities. This study had certain limitations, such as men were underrepresented that prevented us from a complete understanding of the gender-based contribution in the prevalence of hypertension along with the inability to identify their specific needs. Secondly, we could not do an in-depth assessment of dietary habits and physical activity patterns, therefore, findings should be explicated with caution. Furthermore, this was a cross-sectional study, and the identification of causal associations was not possible therefore, future studies are needed to investigate the evolution of hypertension and its management in the same setting.

## Figures and Tables

**Table 1:** Sociodemographic and clinical characteristics of the population along with the status of their blood pressure.

Variables	Total Sample %	Blood Pressure Scores ^1^		
		Normal BP	Hypertension	*p*-Value
**Gender**				
Male	14.9%	34.9%	65.1%	0.061
Female	85.1%	25.1%	74.9 %	
**Age categories**				
18–35	26.0%	37.5%	62.5%	<0.001
36–49	31.4%	33.7%	66.2%	
50–64	30.2%	20.0%	80.0%	
≥65	12.6%	14.9%	85.0%	
**Education**				
None/primary	29.4%	23.9%	76.1%	0.215
High school	52.9%	26.3%	73.7%	
Tertiary	17.7%	33.7%	66.3%	
**Relationship status**				0.984
Single	61.1%	26.5%	73.5%	
In a relationship	38.9%	26.6%	73.4%	
**Employed**				0.948
Yes	43.9%	26.4%	73.6%	
No	56.1%	26.7%	73.3%	
**Monthly income**				
<R1000	58.9%	22.7%	77.3%	0.039
≥R1000	41.1%	30.4%	69.6%	
**Body Mass Index**				
Normal	27.2%	42.9%	57.1%	<0.001
Overweight	25.2%	32.3%	67.1%	
Obese	47.6%	17.1%	82.9%	
**Blood glucose reading**				
Normal range	59.3%	30.8%	69.2%	0.037
Prediabetic range	21.8%	18.8%	81.2%	
Diabetic range	18.8%	24.8%	75.2%	
**Physical activity**				
No (<150 min)	53.5%	25.3	74.7%	0.361
Yes (≥ 150 min)	46.5%	28.7%	71.3%	
**Adherence to Westernized diet**				
Low	11.9%	52.9%	47.1%	<0.001
Moderate	35.1%	28.9%	71.1%	
High	53.1%	18.1%	81.9%	
**Beliefs about hypertension control**				
Hypertension cannot be controlled with medication	84.5%	29.3%	70.6%	0.342
Hypertension can be controlled with diet	22.3%	26.2%	73.7%	0.969

^1^ Chi-square tests of association were conducted; significance is determined at *p* < 0.05 (overall, for a given parameter).

**Table 2:** Multivariable logistic regression model of sociodemographic and clinical factors associated with hypertension.

Variable	aOR ^1^	95% CI	*p*
**Gender: Reference: female**	1		
male	1.18	0.63–2.21	0.609
**Age categories: Reference 18–35**	1		
36–49	0.80	0.46–1.40	0.439
50–64	1.87	1.03–3.39	0.039
≥65	3.20	1.34–7.63	0.009
**Income: Reference < 1000**			
≥1000	1.47	0.95–4.29	0.087
**BMI: Reference normal**			
Normal	1		
overweight	1.53	0.85–2.73	0.149
Obese	3.52	2.01–6.18	<0.001
**Blood glucose: Reference Normal**			
Normal range	1		
Prediabetic range	1.04	0.58–1.85	0.907
Diabetic range	2.24	1.06–4.72	0.034
**Adherence to Westernized diet: Reference Low**			
Low	1		
Moderate	2.94	1.57–5.51	0.001
High	5.35	2.85–10.05	<0.001

^1^ aOR = adjusted odds ratio.

**Table 3:** Sociodemographic and clinical characteristics associated with untreated hypertension among hypertensive participants.

Variables	Bivariate Associations ^1^			Multivariate Associations ^2^		
	Treated Hypertension (43.6%)	Untreated Hypertension (56.4%)	*p*-Value	aOR	95% CI	*p*
**Gender**						
Female	46.9%	53.1%	0.002	1		
Male	19.4%	80.6%		2.93	1.90–7.95	0.034
**Age categories**			<0.001			
18–35	25.0%	75.0%		1		
36–49	34.0%	66.0%		0.85	0.40–1.80	0.666
50–64	52.6%	47.4%		0.45	0.22–0.95	0.035
≥65	66.7%	33.4%		0.19	0.07–0.48	0.000
**Education**						
None/primary	52.4%	47.6%	0.052	1		
High school	37.8%	62.2%		1.57	0.86–2.67	0.140
Tertiary	46.7%	53.3%		1.64	0.68–3.92	0.268
**Relationship status**						
Single	45.3%	54.7%	0.454	-		
In a relationship	41.1%	58.9%				
**Employed**						
yes	38.7%	61.3%	0.127			
no	47.2%	52.8%				
**Monthly income**						
<R1000	42.1%	57.9%	0.474			
≥R1000	46.1%	53.9%				
**Body Mass Index**						
Underweight/normal	25.8%	74.2%	<0.001	1		
Overweight	37.2%	62.8%		0.512	0.23–1.16	0.109
Obese	52.0%	48.0%		0.304	0.15–0.64	0.002
**Blood glucose reading**						
Normal range	40.2%	59.8%	0.163	-		
Prediabetic range	42.9%	57.1%				
Diabetic range	54.1%	45.9%				
**Physical activity**						
No (<150 min)	45.7	54.3%	0.419	-		
Yes (≥150 min)	41.3%	58.7%				
**Adherence to westernized diet**						
Low	63.0%	37.0%	0.106	-		
Moderate	42.2%	57.8%				
High	41.7%	58.3%				
**Beliefs about hypertension control**						
Hypertension cannot be controlled with medication	57.4%	42.6%	0.026	3.02	1.50–6.09	0.002
Hypertension can be controlled with diet	40.0%	60.0%	0.489	-	-	-

^1^ Chi-square tests of association were conducted. ^2^ Multivariable logistic regression, with variables associated with untreated hypertension at *p* < 0.1 entered into the model.
